# Supplementary material for: Does voluntary practice improve the outcome of an OSCE in undergraduate medical studies? A Propensity Score Matching approach
Source: PLoS One. 2024 Oct 24;19(10):e0312387. doi: 10.1371/journal.pone.0312387 (PMC11500970; doi:10.1371/journal.pone.0312387)
Supplement: S1 File — (PDF) [file pone.0312387.s001.pdf]

Does voluntary practice improve the outcome of an OSCE in undergraduate medical studies?
A Propensity Score Matching approach

Supplementary Information

Supplement 1: Sample representativeness tests

Table I-1) SOCIODEMOGRAPHIC VARIABLES

	Study participants	All students	p>T
Female (%)	68.8	65.2	0.292
German (%)	98.0	94.9	0.034
Gymnasium (%)	80.8	79.5	0.647
GPA	1.56	1.55	0.787
	(0.55)	(0.57)	
Waiting time (years)	2.82	2.43	0.103
	(3.71)	(3.23)	
Age	21.94	21.53	0.126
	(3.95)	(3.56)	
**Admission quota:**			
NC (%)	21.95	20.68	0.667
WL/PE (%)	15.45	10.16	0.740
SEL (%)	54.47	53.27	0.021
SP (%)	8.13	15.89	0.002

Notes: Study participants' includes all students who filled out the questionnaire and who consented to connecting their answers with data on study success. 'All students' are all OSCE participants in the years 2021-2023, including the study participants. Statistical significance tested with two-sided t-tests. Standard deviations are in parentheses. Admission quotas: NC ('numerous clausus'): applicants admitted directly based on their excellent GPA. WL/PE ('waiting list'/'professional experience'): applicants admitted via a waiting list or via completed vocational training and a study ability test. SEL ('selection'): university-controlled selection procedure that included interviews until 2019 and a combination of GPA, study ability tests, vocational training or a voluntary service since 2020. SP ('special quotas'): for example international (non-EU) students, military doctors or hardship cases.

Table I-2) OSCE RESULTS

OSCE station	Study participants	All students	p>T
Physical examination (%)	88.23	87.45	0.198
Medical Skills (%)	93.25	91.59	0.003
Neurological examination (%)	84.13	82.57	0.061
Radiology (%)	90.43	87.33	<0.001
Anamnesis (%)	83.50	82.85	0.305
Diagnosis (%)	84.45	82.74	0.010
OSCE overall (%)	87.15	85.75	<0.001

Notes: Study participants' includes all students who filled out the questionnaire and who consented to connecting their answers with data on study success. 'All students' are all OSCE participants in the years 2021-2023, including the study participants. Statistical significance tested with two-sided t-tests. 'Physical examination': average result in stations internal medicine I – internal medicine III. 'Medical skills': average results in stations medical skills I & II. A structured diagnosis of a X-ray ('Radiology') was not offered for Free Practice in the Skills Lab and is thus not part of this study.

Supplement 2:
Additional descriptive statistics and probit regressions

II-1) Sociodemographic indicators and free practice

Table II-1a) FREE PRACTICE AND SOCIODEMOGRAPHIC INDICATORS (t-tests for binary variables)

	Gender			Vocational training			All exams completed		
	male	female	*p*-value	no	yes	*p*-value	no	yes	*p*-value
Free practice (FP)	79.45%	93.57%	<0.001	95.24%	80.39%	<0.001	84.96%	89.58%	0.293
FP (frequency)	2.53	3.54	0.005	3.58	2.72	0.012	2.77	2.79	0.209
Phys. Examination	44.87%	59.06%	0.037	57.14%	50.98%	0.339	61.95%	58.33%	0.384
Neuro. Examination	23.08%	39.77%	0.010	34.01%	35.29%	0.835	46.90%	37.50%	0.802
Anamnesis	17.95%	22.81%	0.387	21.09%	21.57%	0.928	27.43%	35.42%	0.812
Diagnosis	19.23%	23.98%	0.408	23.13%	21.57%	0.773	29.20%	39.58%	0.972
Venipuncture	64.10%	81.87%	0.002	84.35%	64.71%	<0.001	73.45%	72.92%	0.428
ECG	55.13%	58.48%	0.622	59.86%	53.92%	0.353	41.59%	43.80%	0.225
PVC	71.79%	90.64%	<0.001	91.84%	74.51%	<0.002	80.53%	87.50%	0.130
Feeding tube	48.72%	63.16%	0.032	65.99%	48.04%	0.005	38.05%	70.83%	0.702
Rectal examination	32.05%	41.52%	0.156	44.22%	30.39%	0.028	22.12%	39.58%	0.059
Injections	41.03%	58.48%	0.010	62.59%	39.22%	<0.001	43.36%	43.75%	0.555

Notes: Binary sociodemographic control variables. 'Vocational training' = 1 if participants reported professional experience in the medical sector, e.g. as paramedics or trained nurses. 'All exams completed' = 1 if all written and oral exams scheduled before the OSCE were also completed before the OSCE. Statistical significance of group-differences is tested with two-sided t-tests. FP: free practice in the Skills Lab; ECG: electrocardiogram; PVC: peripheral venous catheter.

Table II-1b) FREE PRACTICE & SOCIODEMOGRAPHIC INDICATORS (pairwise correlations for continuous variables)

	School leaving grade	Average exam grade	Age at enrolment
Free practice (FP)	-0.195 (0.002)	-0.269 (0.000)	-0.198 (0.002)
FP (frequency)	-0.235 (0.000)	-0.248 (0.000)	-0.117 (0.066)
Phys. Examination	-0.113 (0.036)	-0.113 (0.077)	-0.019 (0.764)
Neuro. Examination	-0.097 (0.126)	0.023 (0.720)	-0.089 (0.160)
Anamnesis	-0.053 (0.409)	-0.023 (0.726)	-0.030 (0.642)
Diagnosis	-0.069 (0.280)	-0.042 (0.513)	-0.051 (0.427)
Venipuncture	-0.227 (0.003)	-0.182 (0.004)	-0.221 (0.000)
ECG	-0.027 (0.673)	-0.182 (0.004)	-0.112 (0.077)
PVC	-0.207 (0.001)	-0.266 (0.000)	-0.211 (0.001)
Feeding tube	-0.174 (0.006)	-0.202 (0.002)	-0.197 (0.002)
Rectal examination	-0.192 (0.002)	-0.180 (0.005)	-0.169 (0.008)
Injections	-0.233 (0.002)	-0.160 (0.012)	-0.182 (0.004)

Notes: Continuous and categorical control variables. 'School leaving grade' in the range of 100 (best) to 400 (worst passing grade). 'Average exam grade' takes the values 1, 2, 3, and 4, equivalent to an A-D grading system. Pearson correlation coefficients are reported. P-values are in parentheses. FP: free practice in the Skills Lab; ECG: electrocardiogram; PVC: peripheral venous catheter.

II-2) Sociodemographic indicators and OSCE results

Table II-2a) OSCE RESULTS AND SOCIODEMOGRAPHIC INDICATORS (t-tests)

	Gender			Vocational training			All exams completed		
	male	female	*p*-value	no	yes	*p*-value	no	yes	*p*-value
OSCE (%)	86.51	87.44	0.175	87.33	86.88	0.486	85.25	87.51	0.009
Physical exam. (%)	86.65	88.95	0.034	89.41	86.53	0.006	85.13	88.82	0.008
Medical skills (%)	93.03	93.35	0.716	92.81	93.88	0.200	91.54	93.57	0.069
Neurological exam. (%)	84.42	84.00	0.788	84.61	83.44	0.428	79.44	85.02	0.004
Anamnesis & diagnosis (%)	83.08	84.38	0.185	83.62	84.49	0.346	84.33	83.91	0.732

Notes: Binary sociodemographic control variables. 'Vocational training' = 1 if participants reported professional experience in the medical sector, e.g. as paramedics or trained nurses. 'All exams completed' = 1 if all written and oral exams scheduled before the OSCE were also completed before the OSCE. Statistical significance of group-differences is tested with two-sided t-tests. FP: free practice in the Skills Lab; ECG: electrocardiogram; PVC: peripheral venous catheter.

Table II-2b) MEDICAL SKILLS RESULTS AND SOCIODEMOGRAPHIC INDICATORS (*t*-tests)

	Gender			Vocational Training			All exams completed		
	male	female	*p*-value	no	yes	*p*-value	no	yes	*p*-value
Venipuncture (%)	94.92	95.00	0.971	94.69	95.35	0.741	96.00	94.83	0.691
ECG (%)	96.00	91.90	0.263	93.19	92.00	0.683	89.33	93.14	0.379
PVC (%)	92.82	94.33	0.359	92.85	95.27	0.122	92.33	94.16	0.366
Feeding tube (%)	91.24	92.4	0.626	92.10	91.82	0.904	92.5	91.92	0.864
Rectal exam. (%)	90.29	91.23	0.662	91.29	90.35	0.650	91.00	90.93	0.983
Injections (%)	95.85	94.39	0.406	93.71	96.46	0.098	91.79	95.75	0.042

Notes: Binary sociodemographic control variables. 'Vocational training' = 1 if participants reported professional experience in the medical sector, e.g. as paramedics or trained nurses. 'All exams completed' = 1 if all written and oral exams scheduled before the OSCE were also completed before the OSCE. Statistical significance of group-differences is tested with two-sided t-tests. FP: free practice in the Skills Lab; ECG: electrocardiogram; PVC: peripheral venous catheter.

Table II-2c) OSCE RESULTS AND SOCIODEMOPGRAPHIC INDICATORS (pairwise correlations)

	School leaving grade	Avg. Exam grade	Age
OSCE (%)	-0.165	-0.422	-0.092
	(0.010)	(0.000)	(0.150)
Physical examination (%)	-0.174	-0.342	-0.150
	(0.006)	(0.000)	(0.019)
Medical skills (%)	-0.014	-0.155	0.067
	(0.826)	(0.015)	(0.299)
Neurological examination (%)	-0.218	-0.337	-0.142
	(0.001)	(0.000)	(0.026)
Anamnesis and diagnosis (%)	-0.007	-0.189	0.015
	(0.914)	(0.003)	(0.817)

Notes: Continuous and categorical control variables. 'School leaving grade' in the range of 100 (best) to 400 (worst passing grade). 'Average exam grade' takes the values 1, 2, 3, and 4, equivalent to an A-D grading system. Pearson correlation coefficients are reported. P-values are in parentheses. FP: free practice in the Skills Lab; ECG: electrocardiogram; PVC: peripheral venous catheter.

Table II-2d) MEDICAL SKILLS RESULTS AND SOCIODEMOGRAPHIC INICATORS (pairwise correlations)

	School leaving grade	Avg. Exam grade	Age
Venipuncture (%)	0.024	0.009	0.102
	(0.826)	(0.933)	(0.350)
ECG (%)	0.190	0.089	-0.135
	(0.195)	(0.549)	(0.360)
PVC (%)	0.056	-0.180	0.170
	(0.516)	(0.037)	(0.049)
Feeding tube (%)	-0.128	-0.098	-0.057
	(0.324)	(0.454)	(0.664)
Rectal examination (%)	-0.182	-0.116	-0.047
	(0.230)	(0.448)	(0.759)
Injections (%)	0.041	-0.182	0.158
	(0.715)	(0.099)	(0.153)

Notes: Continuous and categorical control variables. 'School leaving grade' in the range of 100 (best) to 400 (worst passing grade). 'Average exam grade' takes the values 1, 2, 3, and 4, equivalent to an A-D grading system. Pearson correlation coefficients are reported. P-values are in parentheses. FP: free practice in the Skills Lab; ECG: electrocardiogram; PVC: peripheral venous catheter.

II-3) Probit regressions for propensity scores

Table II-3a) PROBIT REGRESSIONS FOR PROPENSITY SCORES, PART 1

	(1) Physical examination Coefficients	(2) Physical examination Margins	(3) Neurological examination Coefficients	(4) Neurological examination Margins	(5) Anamnesis Coefficients	(6) Anamnesis Margins	(7) Diagnosis Coefficients	(8) Diagnosis Margins
Vocational training	0.141	0.054	0.318	0.114	0.181	0.053	0.119	0.036
	(0.206)	(0.079)	(0.212)	(0.075)	(0.230)	(0.067)	(0.227)	(0.068)
Abitur grade	-0.003	-0.001	-0.004*	-0.001*	-0.002	-0.001	-0.002	-0.001
	(0.002)	(0.001)	(0.002)	(0.001)	(0.002)	(0.001)	(0.002)	(0.001)
All exams completed	0.006	0.002	0.089	0.032	-0.122	-0.035	-0.105	-0.031
	(0.244)	(0.093)	(0.253)	(0.090)	(0.271)	(0.079)	(0.269)	(0.081)
Average exam grade	-0.177	-0.068	0.081	0.029	-0.061	-0.018	-0.083	-0.025
	(0.129)	(0.049)	(0.132)	(0.047)	(0.145)	(0.042)	(0.144)	(0.043)
Female	0.329	0.126	0.448*	0.160*	0.162	0.047	0.148	0.044
	(0.178)	(0.067)	(0.190)	(0.065)	(0.202)	(0.059)	(0.199)	(0.059)
Observations	245		245		245		245	
Pseudo R^2	0.029		0.034		0.008		0.008	

Notes: Probit regressions with the binary indicator Free Practice (FP) yes/no of Physical examination, Neurological examination, Anamesis, and Diagnosis as dependent variables. Predictors are the five explanatory variables used for the matching. The coefficients in columns (1), (3), (5) and (7) are used to calculated the Propensity Score (PS) in the range [0,1] with Kernel-matching, replacement and a strict requirement of common support. The calculated margins in columns (2), (4), (6) and (8) are not used for matching and are an informative tool: for binary indicatory, the margin gives the likelihood of the dependent variable = 1 (in percentage points) in response of the predictor being = 1. For continuous variables, the response (to a one-unit increase in the explanatory variable) is measured in percent. Standard errors are in parentheses. *: $p<0.05$

Table II-3b) PROBIT REGRESSIONS FOR PROPENSITY SCORES, PART 2

	(1) Venipuncture Coefficients	(2) Venipuncture Margins	(3) ECG Coefficients	(4) ECG Margins	(5) PVC Coefficients	(6) PVC Margins
Vocational training	-0.308	-0.083	0.203	0.074	-0.540	-0.098
	(0.413)	(0.111)	(0.465)	(0.168)	(0.338)	(0.061)
Abitur grade	-0.004	-0.001	0.006	0.002	0.001	0.000
	(0.003)	(0.001)	(0.005)	(0.002)	(0.003)	(0.001)
All exams completed	-0.445	-0.121	-0.439	-0.160	-0.341	-0.062
	(0.481)	(0.128)	(0.642)	(0.230)	(0.435)	(0.079)
Average exam grade	-0.564*	-0.153*	-0.298	-0.109	-0.770*	-0.140*
	(0.252)	(0.063)	(0.323)	(0.114)	(0.265)	(0.046)
Female	0.460	0.125	0.636	0.232	0.872*	0.158*
	(0.341)	(0.089)	(0.562)	(0.197)	(0.316)	(0.054)
Observations	86		48		135	
Pseudo R^2	0.158		0.074		0.202	

Notes: Probit regressions with the binary indicator Free Practice (FP) yes/no of the medical skills Venipuncture, ECG (electrocardiogram) and PVC (peripheral venous catheter) as dependent variables. Predictors are the five explanatory variables used for the matching. The coefficients in columns (1), (3) and (5) are used to calculated the Propensity Score (PS) in the range [0,1] with Kernel-matching, replacement and a strict requirement of common support. The calculated margins in columns (2), (4) and (6) are not used for matching and are an informative tool: for binary indicatory, the margin gives the likelihood of the dependent variable = 1 (in percentage points) in response of the predictor being = 1. For continuous variables, the response (to a one-unit increase in the explanatory variable) is measured in percent. Standard errors are in parentheses. *: $p<0.05$

Table II-3c) PROBIT REGRESSIONS FOR PROPENSITY SCORES, PART 3

	(1) Feeding tube Coefficients	(2) Feeding tube Margins	(3) Rectal examination Coefficients	(4) Rectal examination Margins	(5) Incetions Coefficients	(6) Injections Margins
Vocational training	0.324	0.112	0.406	0.137	-0.117	-0.043
	(0.451)	(0.154)	(0.518)	(0.171)	(0.354)	(0.129)
Abitur grade	-0.006	-0.002	-0.004	-0.001	-0.003	-0.001
	(0.004)	(0.001)	(0.005)	(0.002)	(0.004)	(0.001)
All exams completed	-0.127	-0.044	-1.220	-0.410	-0.010	-0.004
	(0.555)	(0.192)	(0.781)	(0.241)	(0.384)	(0.141)
Average exam grade	-0.089	-0.031	-0.919*	-0.309*	-0.137	-0.050
	(0.290)	(0.100)	(0.366)	(0.095)	(0.224)	(0.081)
Female	0.580	0.201	-0.042	-0.014	0.541	0.198
	(0.366)	(0.118)	(0.484)	(0.163)	(0.308)	(0.106)
Observations	61		45		84	
Pseudo R^2	0.080		0.145		0.058	

Notes: Probit regressions with the binary indicator Free Practice (FP) yes/no of the medical skills insertion of a Feeding tube, digital rectal examination and (intramuscular) injections as dependent variables. Predictors are the five explanatory variables used for the matching. The coefficients in columns (1), (3) and (5) are used to calculated the Propensity Score (PS) in the range [0,1] with Kernel-matching, replacement and a strict requirement of common support. The calculated margins in columns (2), (4) and (6) are not used for matching and are an informative tool: for binary indicatory, the margin gives the likelihood of the dependent variable = 1 (in percentage points) in response of the predictor being = 1. For continuous variables, the response (to a one-unit increase in the explanatory variable) is measured in percent. Standard errors are in parentheses. *: $p<0.05$

Supplement 3:
Quality of matching and robustness checks

III-1) Propensity score tests

The **Tables III-1a – III-1j** give, for each examined part of the OSCE, the composition of the groups FP ('Free Practice', i.e., the treatment group) and No FP (the control group) pre (U) and post (M) matching in each of the five sociodemographic variables used for matching. 'Bias (%)' refers to the difference between the groups FP and No FP, e.g. in the share of participants reporting vocational training. 'Reduced bias (%)' shows how propensity score matching affected the composition of treatment and control group. A negative value in 'Reduced bias (%)' means that matching introduced or strengthened group differences in a respective sociodemographic factor. This may happen as the result of the matching algorithm to reduce overall bias over all variables used for matching. Table 3 in the main text shows overall bias pre and post matching for all outcome variables used in this study.
The statistical significance of group differences in **Tables III-1** is tested with two-sided *t*-tests.

Table III-1a) PS-TESTS PHYSICAL EXAMINATION

Variable	Matching status	Mean		Bias (%)	Reduced bias (%)	*t*-test	
		FP	No FP			|T|	*p*>T
Vocational training (%)	U	38.52	43.53	-10.4		-0.81	0.420
	M	37.59	37.39	0.4	96.2	0.03	0.974
School leaving grade (Abitur)	U	149.56	164.18	-26.5		-2.08	0.039
	M	149.85	150.25	-0.7	97.3	-0.07	0.948
All exams completed (%)	U	85.93	81.81	11.1		0.87	0.384
	M	85.71	85.55	0.5	95.2	0.05	0.963
Average grade in exams	U	2.09	2.25	-22.7		-1.78	0.077
	M	2.11	2.12	-1.9	91.6	-0.16	0.872
Female (%)	U	74.07	62.72	24.5		1.92	0.057
	M	73.68	73.32	0.8	96.8	0.07	0.946

Notes: U: unmatched; M: matched. FP: free practice

Table III-1b) PS-TESTS NEUROLOGICAL EXAMINATION

Variable	Matching status	Mean		Bias (%)	Reduced bias (%)	*t*-test	
		FP	No FP			|T|	*p*>T
Vocational training (%)	U	41.86	40.25	3.3		0.24	0.808
	M	41.86	39.38	5.0	-54.4	0.33	0.742
School leaving grade (Abitur)	U	148.95	160	-20.7		-1.50	0.135
	M	148.95	148.97	-0.0	99.8	-0.00	0.998
All exams completed (%)	U	84.88	83.65	3.4		0.25	0.802
	M	84.88	84.47	1.1	66.6	0.07	0.941
Average grade in exams	U	2.19	2.15	4.9		0.36	0.720
	M	2.19	2.20	-1.7	66.1	-0.11	0.915
Female (%)	U	79.07	63.52	34.7		2.53	0.012
	M	79.07	78.92	0.3	99.0	0.02	0.980

Notes: U: unmatched; M: matched. FP: free practice

Table III-1c) PS-TESTS ANAMNESIS

Variable	Matching status	Mean FP	Mean No FP	Bias (%)	Reduced Bias (%)	*t*-test |T|	*t*-test *p*>T
Vocational training	U	41.51	40.63	1.8		0.12	0.908
(%)	M	40.39	37.43	6.0	-234.4	0.31	0.760
School leaving	U	150.75	157.6	-12.9		-0.80	0.424
grade (Abitur)	M	151.54	150.41	2.1	83.5	0.12	0.908
All exams comple-	U	83.02	84.38	-3.6		-0.24	0.812
ted (%)	M	84.62	84.04	1.6	57.3	0.08	0.936
Average grade in	U	2.13	2.17	-5.6		-0.35	0.726
exams	M	2.12	2.15	-5.5	1.3	-0.28	0.780
Female (%)	U	73.59	67.71	12.9		0.82	0.415
	M	73.08	72.96	0.3	98.0	0.01	0.989

Notes: U: unmatched; M: matched. FP: free practice

Table III-1d) PS-TESTS DIAGNOSIS

Variable	Matching status	Mean FP	Mean No FP	Bias (%)	Reduced Bias (%)	*t*-test |T|	*t*-test *p*>T
Vocational training	U	39.29	41.27	-4.0		-0.26	0.792
(%)	M	39.29	38.21	2.2	45.8	0.12	0.908
School leaving	U	149.29	158.15	-16.6		-1.06	0.291
grade (Abitur)	M	149.29	149.45	-0.3	98.1	-0.02	0.986
All exams comple-	U	83.93	84.13	-0.5		-0.04	0.972
ted (%)	M	83.93	83.72	0.6	-7.9	0.03	0.976
Average grade in	U	2.11	2.18	-10.2		-0.66	0.513
exams	M	2.11	2.15	-6.5	36.8	-0.34	0.732
Female (%)	U	73.21	67.73	12.0		0.78	0.437
	M	73.21	72.73	1.1	91.2	0.06	0.954

Notes: U: unmatched; M: matched. FP: free practice

Table III-1e) PS-TESTS MEDICAL SKILL: VENIPUNCTURE

Variable	Matching status	Mean FP	Mean No FP	Bias (%)	Reduced Bias (%)	*t*-test |T|	*t*-test *p*>T
Vocational training	U	34.92	65.22	-62.6		-2.58	0.012
(%)	M	52.94	47.33	11.6	81.5	0.46	0.650
School leaving	U	142.06	183.04	-68.4		-3.06	0.003
grade (Abitur)	M	160	158.58	2.4	96.5	0.11	0.912
All exams comple-	U	87.30	86.96	1.0		0.04	0.967
ted (%)	M	82.35	90.29	-23.3	-2198.9	-0.94	0.349
Average grade in	U	1.94	2.39	-70.8		-2.66	0.009
exams	M	2.38	2.27	17.0	76.0	0.84	0.402
Female (%)	U	74.60	56.52	38.1		1.62	0.109
	M	67.65	62.48	10.9	71.4	0.44	0.660

Notes: U: unmatched; M: matched. FP: free practice

Table III-1f) PS-TESTS MEDICAL SKILL: ECG

Variable	Matching status	Mean FP	Mean No FP	Bias (%)	Reduced Bias (%)	*t*-test |T|	*t*-test *p*>T
Vocational training (%)	U	53.85	31.82	44.7		1.54	0.131
	M	40.00	33.24	13.7	69.3	0.43	0.667
School leaving grade (Abitur)	U	168.46	144.55	44.6		1.52	0.136
	M	147	147.88	-1.6	96.3	-0.06	0.951
All exams completed (%)	U	84.62	90.91	-18.9		-0.65	0.521
	M	85.00	87.60	-7.8	58.8	-0.23	0.817
Average grade in exams	U	2	2	0.0		-0.00	1.000
	M	1.95	1.95	0.5		0.02	0.987
Female (%)	U	84.62	77.27	18.4		0.64	0.526
	M	80.00	82.72	-6.8	62.9	-0.22	0.830

Notes: U: unmatched; M: matched. FP: free practice

Table III-1g) PS-TESTS MEDICAL SKILL: PVC

Variable	Matching status	Mean FP	Mean No FP	Bias (%)	Reduced Bias (%)	*t*-test |T|	*t*-test *p*>T
Vocational training (%)	U	36.21	68.42	-67.1		-2.70	0.008
	M	46.67	36.00	22.2	66.9	1.45	0.148
School leaving grade (Abitur)	U	156.47	180.53	-42.8		-1.80	0.074
	M	162.22	184.94	-40.4	5.6	-2.43	0.016
All exams completed (%)	U	83.62	73.68	24.0		1.05	0.297
	M	81.11	81.02	0.2	99.1	0.02	0.987
Average grade in exams	U	2.16	2.74	-70.9		-3.30	0.001
	M	2.32	2.39	-7.8	88.9	-0.64	0.523
Female (%)	U	71.55	42.11	61.2		2.58	0.011
	M	63.33	62.02	2.7	95.5	0.18	0.856

Notes: U: unmatched; M: matched. FP: free practice. PVC: peripheral venous catheter.

Table III-1h) PS-TESTS MEDICAL SKILL: FEEDING TUBE

Variable	Matching status	Mean FP	Mean No FP	Bias (%)	Reduced Bias (%)	*t*-test |T|	*t*-test *p*>T
Vocational training (%)	U	34.21	39.13	-10.0		-0.38	0.704
	M	31.03	36.29	-10.7	-6.8	-0.42	0.678
School leaving grade (Abitur)	U	144.47	171.3	-45.7		-1.79	0.078
	M	152.41	158.74	-10.8	76.4	-0.45	0.657
All exams completed (%)	U	86.84	86.96	-0.3		-0.01	0.990
	M	89.66	86.46	9.3	-2690.8	0.37	0.714
Average grade in exams	U	2.11	2.22	-16.0		-0.60	0.551
	M	2.31	2.12	27.1	-69.4	1.08	0.283
Female (%)	U	73.68	52.17	44.9		1.73	0.089
	M	65.52	65.30	0.4	99.0	0.02	0.987

Notes: U: unmatched; M: matched. FP: free practice

Table III-1i) PS-TESTS MEDICAL SKILL: RECTAL EXAMINATION

Variable	Matching status	Mean FP	Mean No FP	Bias (%)	Reduced Bias (%)	*t*-test |T|	*t*-test *p*>T
Vocational training (%)	U	36.36	39.13	-5.6		-0.19	0.852
	M	38.46	26.51	24.1	-332.1	0.63	0.534
School leaving grade (Abitur)	U	148.18	156.09	-13.5		-0.45	0.652
	M	150	141.41	14.7	-8.7	0.41	0.687
All exams completed (%)	U	86.36	95.65	-32.2		-1.08	0.284
	M	92.31	97.71	-18.7	41.9	-0.61	0.546
Average grade in exams	U	2	2.43	-71.8		-2.42	0.020
	M	2.31	2.23	12.8	82.2	0.43	0.672
Female (%)	U	68.18	69.57	-2.9		-0.10	0.922
	M	69.23	67.31	4.0	-38.5	0.10	0.920

Notes: U: unmatched; M: matched. FP: free practice

Table III-1j) PS-TESTS MEDICAL SKILL: INJECTIONS

Variable	Matching status	Mean FP	Mean No FP	Bias (%)	Reduced Bias (%)	*t*-test |T|	*t*-test *p*>T
Vocational training (%)	U	34.69	51.43	-33.9		-1.54	0.128
	M	34.69	35.98	-2.6	92.3	-0.13	0.895
School leaving grade (Abitur)	U	147.76	165.43	-35.3		-1.62	0.108
	M	147.76	149.58	-3.6	89.7	-0.19	0.849
All exams completed (%)	U	79.59	74.29	12.5		0.57	0.572
	M	79.59	79.09	1.2	90.5	0.06	0.951
Average grade in exams	U	2.14	2.34	-26.0		-1.18	0.240
	M	2.14	2.13	1.4	94.7	0.07	0.945
Female (%)	U	75.51	57.14	39.1		1.79	0.077
	M	75.51	76.60	-2.3	94.1	-0.12	0.901

Notes: U: unmatched; M: matched. FP: free practice

III-2) Propensity score graphs

The histograms in Figure III-2 show, for each part of the OSCE, the frequency of propensity scores for the control group ('No FP', gray bars), the treatment group ('FP', black bars) with matched counterparts in the control group, and subjects with FP who did not have a matched partner and were thus excluded from the analysis.

Generally, when propensity scores lie in the same range and are comparably frequent, subjects are fairly similar with respect to the covariates used for matching. In our case this is in particular the case with larger number of observations, e.g. panels a) – d) in Fig. III-2.

Fig. III-2 is in essence a graphical representation of Tables III-1 in this Attachment and of Table 3 in the main text.

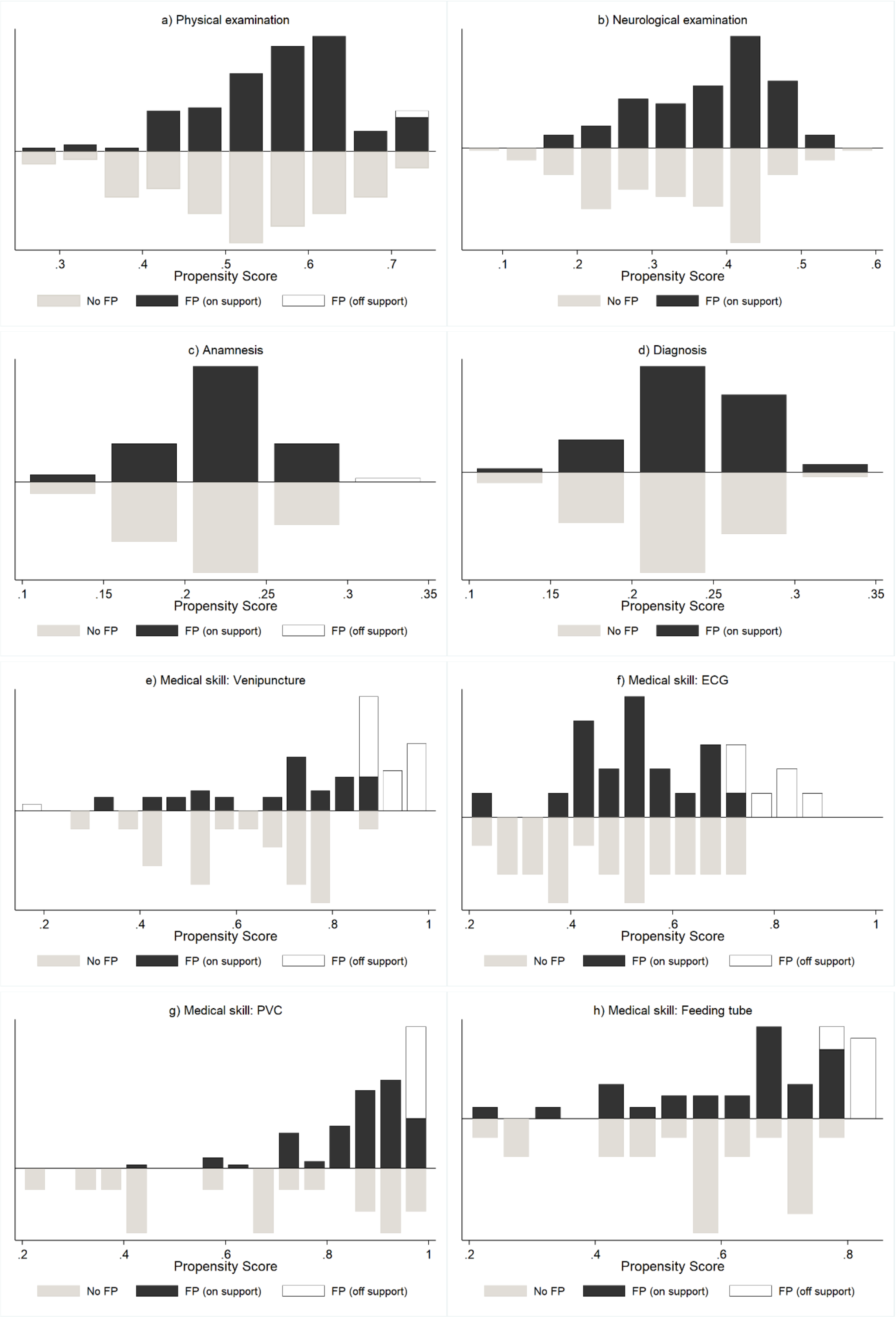
a) Physical examination
.3 .4 .5 .6 .7
Propensity Score
No FP
FP (on support)
FP (off support)
b) Neurological examination
.1 .2 .3 .4 .5 .6
Propensity Score
No FP
FP (on support)
c) Anamnesis
.1 .15 .2 .25 .3 .35
Propensity Score
No FP
FP (on support)
FP (off support)
d) Diagnosis
.1 .15 .2 .25 .3 .35
Propensity Score
No FP
FP (on support)
e) Medical skill: Venipuncture
.2 .4 .6 .8 1
Propensity Score
No FP
FP (on support)
FP (off support)
f) Medical skill: ECG
.2 .4 .6 .8 1
Propensity Score
No FP
FP (on support)
FP (off support)
g) Medical skill: PVC
.2 .4 .6 .8 1
Propensity Score
No FP
FP (on support)
FP (off support)
h) Medical skill: Feeding tube
.2 .4 .6 .8
Propensity Score
No FP
FP (on support)
FP (off support)

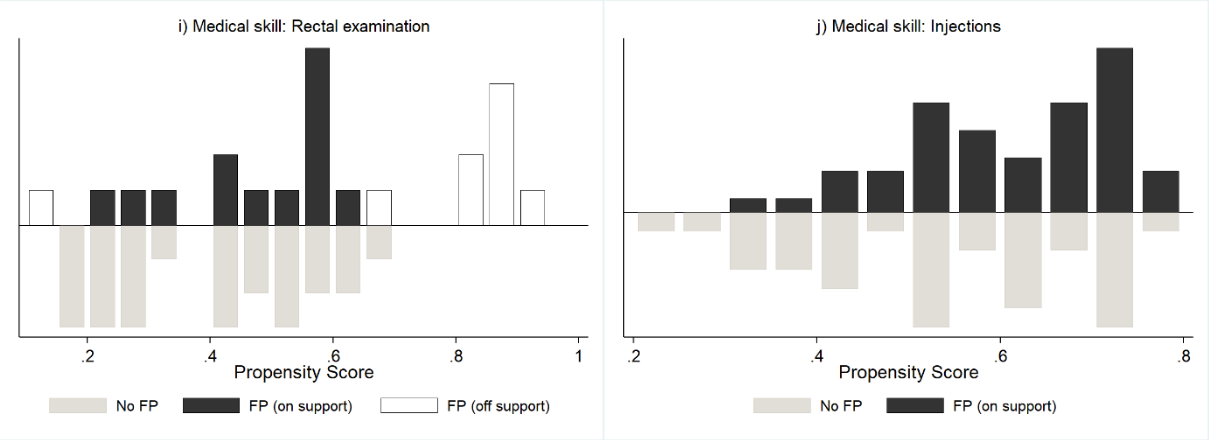

Figure III-2) PROPENSITY SCORE GRAPHS

III-3) Rosenbaum-Bounds

The aim of Propensity Score Matching is to draw causal inferences from observational data. However, even after a successful matching, this is connected to a statistical uncertainty that is hard to quantify. In our study, we balanced some factors we identified as potential confounders, but could not include others, e.g. because they were unobservable to us. Thus, potential selection biases in the data could only be partially reduced.
With sensitivity analyses in this section we model how strongly unobserved covariates can be biased before the treatment effects we identified with PSM (see Table 2 in the main text) become insignificant.
In **Tables III-3**, we use Rosenbaum-Bounds (Rosenbaum, 2002; DiPrete & Gangl, 2004) to test the robustness of treatment effects. Γ (Gamma) in the tables is the Odds-Ratio of subjects‘ allocation to the treatment (FP) and control group (No FP), which is influenced by observed and unobserved characteristics. A Γ of 1 is the equivalent of a randomizes sample, i.e. where there is no difference between treatment and control group and where there is no confounding. Thus, the larger Γ, the larger the modeled imbalance between treatment and control group (Müller, 2012). A treatment effect is 'robust' if the null hypothesis (assuming that the entire effect is the result of confounding) cannot be rejected even for larger Γ. Aakvik (2001) described significant treatment effects with a Γ of 2 as very robust.
In the Tables III-3 we test the robustness of the ATT (Average treatment effect on the treated) from our PSM (see Table 2 in the main text) up to Γ=2. Of major importance and indicated in bold letters is the upper-bound significance level (sig+), which tests positive selection bias and thus an over-estimation of treatment effects.
In contrast to PSM, Rosenbaum-Bounds are median-based. Thus, the significance level in Tables III-3 and Table 2 in the main text can deviate (Mueller, 2012).

With respect to robustness, the treatment effects in our study fall in three broad categories.
First, for some OSCE-stations and skills, FP has a large effect on OSCE performance even when we model large confounding. These are 'Physical examination' (Table III-3a), PVC (III-

3g), rectal examination (III-3i) and injections (III-3j). Second, there are OSCE stations in which FP has no effect on exam success regardless how equal or unequal treatment and control group are. These cases are ‘Neurological examination’, ‘Anamnesis’ and ‘Diagnosis’ (Tables III-3b-d). Third, for the medical skills ‘Venipuncture’ and ‘ECG’ treatment effects become significant only when large confounding is modeled. This suggests that the small observed differences between FP and No FP are explained by unobserved factors we consequentially could not use for matching.

Overall, the sensitivity checks imply that our matching is statistically robust, because mostly positive treatment effects either remain significant or never become significant.

Table III-3a) ROSENBAUM-BOUNDS PHYSICAL EXAMINATION

Γ	sig+	sig-	t-hat+	t-hat-	CI+	CI-
**1**	**0.0000**	0.0000	5.3943	5.3943	4.0753	6.6145
**1.1**	**0.0000**	0.0000	5.1048	5.7000	3.7653	6.9245
**1.2**	**0.0000**	0.0000	4.8184	5.9455	3.4323	7.1785
**1.3**	**0.0000**	0.0000	4.5257	6.2328	3.1131	7.4118
**1.4**	**0.0000**	0.0000	4.2737	6.4315	2.8079	7.6524
**1.5**	**0.0000**	0.0000	4.0373	6.6417	2.5638	7.8452
**1.6**	**0.0000**	0.0000	3.8534	6.8373	2.3492	8.0202
**1.7**	**0.0000**	0.0000	3.6468	7.0378	2.0658	8.2439
**1.8**	**0.0000**	0.0000	3.4395	7.1785	1.8196	8.3897
**1.9**	**0.0002**	0.0000	3.2218	7.3209	1.6212	8.5327
**2**	**0.0004**	0.0000	3.0517	7.4667	1.4279	8.6470

Notes: Γ: log odds of differential assignment due to unobserved factors; sig+: upper bound significance level; sig-: lower bound significance level; t-hat+: upper bound Hodges-Lehman point estimate; t-hat-: lower bound Hodges-Lehman point estimate; CI+: upper bound 95%-confidence interval; CI-: lower bound 95%-confidence interval.

Table III-3b) ROSENBAUM-BOUNDS NEUROLOGICAL EXAMINATION

Γ	sig+	sig-	t-hat+	t-hat-	CI+	CI-
**1**	**0.0259**	0.0259	2.2074	2.207	-0.0279	4.8076
**1.1**	**0.0591**	0.0099	1.6493	2.6224	-0.4439	5.2360
**1.2**	**0.1118**	0.0036	1.2696	3.1702	-0.8581	5.7096
**1.3**	**0.1837**	0.0013	0.9203	3.6204	-1.2899	6.1412
**1.4**	**0.2708**	0.0004	0.6095	4.0108	-1.6446	6.4914
**1.5**	**0.3669**	0.0001	0.3782	4.3897	-1.9951	6.7442
**1.6**	**0.4650**	0.0000	0.0739	4.6750	-2.4992	7.1136
**1.7**	**0.5593**	0.0000	-0.2251	4.9601	-2.8829	7.4172
**1.8**	**0.6453**	0.0000	-0.3999	5.1866	-3.2441	7.7034
**1.9**	**0.7205**	0.0000	-0.6207	5.4661	-3.5110	7.9579
**2**	**0.7839**	0.0000	-0.8581	5.7119	-3.9376	8.2271

Notes: Γ: log odds of differential assignment due to unobserved factors; sig+: upper bound significance level; sig-: lower bound significance level; t-hat+: upper bound Hodges-Lehman point estimate; t-hat-: lower bound Hodges-Lehman point estimate; CI+: upper bound 95%-confidence interval; CI-: lower bound 95%-confidence interval.

Table III-3c) ROSENBAUM-BOUNDS ANAMNESIS

Γ	sig+	sig-	t-hat+	t-hat-	CI+	CI-
1	**0.2589**	0.25895	0.8352	0.8352	-1.9401	3.4530
1.1	**0.3638**	0.1720	0.5304	1.1853	-2.4182	3.7509
1.2	**0.4695**	0.1108	0.1127	1.5201	-2.8082	4.0381
1.3	**0.5688**	0.0698	-0.2384	1.9380	-3.1935	4.3023
1.4	**0.6572**	0.0430	-0.4809	2.2022	-3.5720	4.6008
1.5	**0.7327**	0.0262	-0.7845	2.5389	-3.8815	4.9574
1.6	**0.7949**	0.0157	-1.0546	2.7485	-4.1797	5.2428
1.7	**0.8449**	0.0093	-1.4182	2.9341	-4.4852	5.5129
1.8	**0.8842**	0.0055	-1.6808	3.1965	-4.7905	5.8344
1.9	**0.9144**	0.0032	-1.9366	3.4232	-5.0241	6.0391
2	**0.9373**	0.0018	-2.1682	3.5674	-5.2486	6.2960

Notes: Γ: log odds of differential assignment due to unobserved factors; sig+: upper bound significance level; sig-: lower bound significance level; t-hat+: upper bound Hodges-Lehman point estimate; t-hat-: lower bound Hodges-Lehman point estimate; CI+: upper bound 95%-confidence interval; CI-: lower bound 95%-confidence interval.

Table III-3d) ROSENBAUM-BOUNDS DIAGNOSIS

Γ	sig+	sig-	t-hat+	t-hat-	CI+	CI-
1	**0.2623**	0.2623	0.8100	0.8100	-1.6586	2.9268
1.1	**0.3720**	0.1717	0.1631	1.0193	-1.9816	3.1151
1.2	**0.4821**	0.1088	0.0382	1.2954	-2.1876	3.6057
1.3	**0.5850**	0.0671	-0.1292	1.7600	-2.8291	3.8556
1.4	**0.6755**	0.0404	-0.4192	1.9261	-2.9476	4.0683
1.5	**0.7515**	0.0234	-0.8768	2.0742	-3.1038	4.2954
1.6	**0.8132**	0.0140	-1.0561	2.2954	-3.2524	4.6390
1.7	**0.8618**	0.008	-1.1454	2.7669	-3.6695	4.8870
1.8	**0.8992**	0.0046	-1.3343	2.8816	-3.9714	5.0085
1.9	**0.9273**	0.0026	-1.7524	2.9634	-4.1163	5.1381
2	**0.9481**	0.0015	-1.9285	3.0765	-4.2217	5.5088

Notes: Γ: log odds of differential assignment due to unobserved factors; sig+: upper bound significance level; sig-: lower bound significance level; t-hat+: upper bound Hodges-Lehman point estimate; t-hat-: lower bound Hodges-Lehman point estimate; CI+: upper bound 95%-confidence interval; CI-: lower bound 95%-confidence interval.

Table III-3e) ROSENBAUM-BOUNDS VENIPUNCTURE

Γ	sig+	sig-	t-hat+	t-hat-	CI+	CI-
1	**0.1892**	0.1892	-1.6970	-1.6970	-5.6970	1.7878
1.1	**0.1309**	0.2605	-2.2024	-0.8822	-6.000	1.9115
1.2	**0.0894**	0.3355	-2.7898	-0.4175	-6.3909	2.6946
1.3	**0.0604**	0.4107	-3.0373	-0.2622	-6.8271	2.8239
1.4	**0.0405**	0.4833	-3.5615	0.0000	-8.0000	3.0151
1.5	**0.0269**	0.5514	-4.2122	0.3030	-8.2024	3.2170
1.6	**0.0178**	0.6139	-4.2122	0.7102	-8.2622	3.2252
1.7	**0.0118**	0.6701	-4.3909	1.1080	-8.4592	3.3934
1.8	**0.0077**	0.7120	-4.3915	1.2102	-8.8598	3.3963
1.9	**0.0051**	0.7636	-4.5432	1.2873	-9.6713	3.5132
2	**0.0033**	0.8014	-4.7127	1.6085	-10.000	3.5727

Notes: Γ: log odds of differential assignment due to unobserved factors; sig+: upper bound significance level; sig-: lower bound significance level; t-hat+: upper bound Hodges-Lehman point estimate; t-hat-: lower bound Hodges-Lehman point estimate; CI+: upper bound 95%-confidence interval; CI-: lower bound 95%-confidence interval.

Table III-3f) ROSENBAUM-BOUNDS ECG

Γ	sig+	sig-	t-hat+	t-hat-	CI+	CI-
1	**0.3203**	0.3203	-1.0591	-1.0591	-7.7543	4.6514
1.1	**0.2568**	0.3892	-1.9901	-0.9923	-8.4992	4.9985
1.2	**0.2047**	0.4552	-2.5105	-0.2078	-8.9444	5.4467
1.3	**0.1625**	0.5169	-3.2767	0.1620	-9.9974	6.1216
1.4	**0.1286**	0.5739	-3.8125	0.23239	-10.527	6.3933
1.5	**0.1015**	0.6256	-4.0347	0.91324	-11.243	6.7446
1.6	**0.0800**	0.6721	-4.4469	1.4387	-11.277	7.4387
1.7	**0.0629**	0.7137	-4.5613	1.4895	-11.474	7.6322
1.8	**0.0494**	0.7506	-4.9632	1.6326	-12.000	7.7788
1.9	**0.0388**	0.7832	-4.9985	2.1753	-12.491	8.3060
2	**0.0304**	0.8118	-5.1267	2.2613	-12.713	8.8774

Notes: Γ: log odds of differential assignment due to unobserved factors; sig+: upper bound significance level; sig-: lower bound significance level; t-hat+: upper bound Hodges-Lehman point estimate; t-hat-: lower bound Hodges-Lehman point estimate; CI+: upper bound 95%-confidence interval; CI-: lower bound 95%-confidence interval.

Table III-3g) ROSENBAUM-BOUNDS PVC

Γ	sig+	sig-	t-hat+	t-hat-	CI+	CI-
1	**0.0000**	0.0000	11.019	11.019	9.1447	12.6334
1.1	**0.0000**	0.0000	10.7025	11.3875	8.7222	12.9491
1.2	**0.0000**	0.0000	10.4639	11.6986	8.3795	13.2788
1.3	**0.0000**	0.0000	10.104	11.9867	8.0309	13.5494
1.4	**0.0000**	0.0000	9.8117	12.2215	7.7452	13.7834
1.5	**0.0000**	0.0000	9.522	12.4538	7.3714	14.0576
1.6	**0.0000**	0.0000	9.1794	12.5765	6.9698	14.2554
1.7	**0.0000**	0.0000	8.9548	12.7652	6.6855	14.4481
1.8	**0.0000**	0.0000	8.7222	12.9578	6.5200	14.5825
1.9	**0.0000**	0.0000	8.5653	13.1447	6.1831	14.7652
2	**0.0000**	0.0000	8.3292	13.3124	5.9764	14.8854

Notes: Γ: log odds of differential assignment due to unobserved factors; sig+: upper bound significance level; sig-: lower bound significance level; t-hat+: upper bound Hodges-Lehman point estimate; t-hat-: lower bound Hodges-Lehman point estimate; CI+: upper bound 95%-confidence interval; CI-: lower bound 95%-confidence interval. PVC: Peripheral venous catheter.

Table III-3h) ROSENBAUM-BOUNDS FEEDING TUBE

Γ	sig+	sig-	t-hat+	t-hat-	CI+	CI-
1	**0.0245**	0.0245	3.8368	3.8368	0.0000	6.1225
1.1	**0.0404**	0.0141	3.6059	3.9486	-0.2899	6.4545
1.2	**0.0609**	0.0081	3.1237	4.3008	-0.5060	6.8269
1.3	**0.0857**	0.0046	2.5961	4.4444	-0.6473	7.1237
1.4	**0.1146**	0.0026	2.1309	4.6184	-0.8296	7.5789
1.5	**0.1467**	0.0015	1.8506	4.7845	-0.9413	7.8369
1.6	**0.1815**	0.0009	1.4311	4.9135	-1.0833	8.0264
1.7	**0.2183**	0.0005	1.3119	5.0100	-1.1985	8.3500
1.8	**0.2564**	0.0003	1.1075	5.1298	-1.3029	8.7258
1.9	**0.2955**	0.0002	0.8901	5.3285	-1.4649	8.9090
2	**0.3343**	0.0001	0.7785	5.4557	-1.5614	9.0539

Notes: Γ: log odds of differential assignment due to unobserved factors; sig+: upper bound significance level; sig-: lower bound significance level; t-hat+: upper bound Hodges-Lehman point estimate; t-hat-: lower bound Hodges-Lehman point estimate; CI+: upper bound 95%-confidence interval; CI-: lower bound 95%-confidence interval.

Table III-3i) ROSENBAUM-BOUNDS RECTAL EXAMINATION

**Γ**	**sig+**	sig-	t-hat+	t-hat-	CI+	CI-
**1**	**0.0095**	0.0095	4.1764	4.1764	0.1763	8.6020
**1.1**	**0.0141**	0.0062	4.1764	4.3059	0.1764	8.8985
**1.2**	**0.0195**	0.0041	4.1549	4.4302	0.1764	9.0276
**1.3**	**0.0259**	0.0027	3.8254	4.6020	-0.1746	9.3559
**1.4**	**0.0329**	0.0018	3.4271	4.8558	-0.1746	10.2779
**1.5**	**0.0407**	0.0012	3.1802	5.5344	-0.2524	10.6059
**1.6**	**0.0497**	0.0008	2.9309	5.6161	-0.5256	11.0315
**1.7**	**0.0579**	0.0005	2.9264	5.7197	-1.6230	11.6485
**1.8**	**0.0672**	0.0003	2.9264	5.9814	-2.7487	12.877
**1.9**	**0.0769**	0.0002	2.5754	6.2549	-3.0024	12.8985
**2**	**0.0868**	0.0002	1.8096	6.6059	-3.0024	13.0354

Notes: Γ: log odds of differential assignment due to unobserved factors; sig+: upper bound significance level; sig-: lower bound significance level; t-hat+: upper bound Hodges-Lehman point estimate; t-hat-: lower bound Hodges-Lehman point estimate; CI+: upper bound 95%-confidence interval; CI-: lower bound 95%-confidence interval.

Table III-3j) ROSENBAUM-BOUNDS INJECTIONS

**Γ**	**sig+**	sig-	t-hat+	t-hat-	CI+	CI-
**1**	**0.0000**	0.0000	6.7427	6.7427	5.4343	8.0664
**1.1**	**0.0000**	0.0000	6.6088	6.8715	5.1641	8.2110
**1.2**	**0.0000**	0.0000	6.4721	7.0875	5.0516	8.3029
**1.3**	**0.0000**	0.0000	6.3660	7.2832	4.7406	8.3783
**1.4**	**0.0000**	0.0000	6.1833	7.4931	4.6221	8.4704
**1.5**	**0.0000**	0.0000	6.0578	7.5427	4.4535	8.6457
**1.6**	**0.0000**	0.0000	5.9188	7.6721	4.3882	8.7914
**1.7**	**0.0000**	0.0000	5.7877	7.7257	4.0469	8.8864
**1.8**	**0.0000**	0.0000	5.7056	7.8776	3.7877	9.0217
**1.9**	**0.0001**	0.0000	5.4931	8.0072	3.6944	9.1572
**2**	**0.0001**	0.0000	5.3663	8.0953	3.4559	9.2386

Notes: Γ: log odds of differential assignment due to unobserved factors; sig+: upper bound significance level; sig-: lower bound significance level; t-hat+: upper bound Hodges-Lehman point estimate; t-hat-: lower bound Hodges-Lehman point estimate; CI+: upper bound 95%-confidence interval; CI-: lower bound 95%-confidence interval.

References

AAKVIK, A. (2001) Bounding a matching estimator: The case of a Norwegian training program. OXFORD BULLETIN OF ECONOMICS AND STATISTICS 63: 115-143.

DiPRETE, T.A. & GANGL, M. (2004) Assessing bias in the estimation of causal effects: Rosenbaum bounds on matching estimators and instrumental variables estimation with imperfect instruments. SOCIOLOGICAL METHODOLOGY 34: 271-310.

ROSENBAUM, P.R. (2002) Observational studies. New York: Springer (2nd ed.)

MÜLLER, C.E. (2012). Quasiexperimentelle Wirkungsevaluation mit Propensity Score Matching: ein Leitfaden für die Umsetzung mit Stata. CEVaL-ARBEITSPAPIER 19.

Supplement 4: English translation of the questionnaire

The following pages show the English translation of all items included in the 2023 version of the online-questionnaire sent to students. The original, German online version was distributed via Email.
Questionnaires in earlier years varied slightly, e.g. because the items polling for Free Practice outside the Skills Lab were not yet included.
Not all items included in the questionnaire were ultimately utilized/analyzed for this study.

1. Introduction

Dear student. With this short questionnaire we would like to record your motives for using the MHH Skills Lab and your competencies in certain practical skills. In order to optimize the Skills Lab and adapt it to your needs, we would like to link your information and experiences with your results in the upcoming OSCE. We will ask for your consent at the end of the questionnaire.
Your data will be anonymized by the Office of the Dean of Studies, used exclusively for research purposes and not passed on to third parties. The data will only be processed by the Office of the Dean of Studies and will not be passed on to the lecturers or teaching staff involved.
Even if you did not visit the Skills Lab for Free Practice, your information is very valuable! Thank you for your cooperation!

2. Free Practice in the Summer Term 2023

2.1 Have you used the Skills Lab for Free Practice in this summer term?
(yes/no)

2.2 How often have you used the Skills Lab for Free Practice in this summer term?
(once, twice, 3, 4, …, 10, >10 times)
Question only activated when the answer to 2.1 was (yes)

2.3 What have you practiced on these occasions? (Multiple responses possible)
(venipuncture, ECG diagnosis, physical examination, conducting an anamnesis, neurological examination, conveying a diagnosis, placement of a peripheral venous catheter, placement of a feeding tube, digital-rectal examination, subcutaneous/intramuscular injection, other)
Question only activated when the answer to 2.1 was (yes)

2.4 Please specify “other”.
(Text box)
Question only activated if the box “other” was ticked in question 2.3

2.5 Have you practiced one or several of the following skills in other places than the Skills Lab (e.g. with friends or family)? (Multiple responses possible)
(physical examination, neurological examination, conducting an anamnesis, conveying a diagnosis)

2.6 Here you can give details where and with whom you practiced outside the Skills Lab.
(Text box)
Question only activated if one of the boxes in 2.5 was checked.

2.7 How often have you practiced these skills outside the Skills Lab?
(once, twice, 3, 4, …, 10, >10 times)
Question only activated if one of the boxes in 2.5 was checked.

3. Free practice in earlier semesters

3.1 Have you used the Skills Lab for Free Practice once or several times before this summer term?
(yes/no)

3.2 3.2 How often have you used the Skills Lab for Free Practice before this summer term?
(once, twice, 3, 4, …, 10, >10 times)
Question only activated when the answer to 3.1 was (yes)

3.3 What have you practiced on these occasions? (Multiple responses possible)
(venipuncture, ECG diagnosis, physical examination, conducting an anamnesis, neurological examination, conveying a diagnosis, placement of a peripheral venous catheter, placement of a feeding tube, digital-rectal examination, subcutaneous/intramuscular injection, other)
Question only activated when the answer to 3.1 was (yes)

3.4 Please specify "other".
(Text box)
Question only activated if the box "other" was ticked in question 3.3

4. Information on the use of the Skills Lab

Why do you use the Skills Lab for Free Practice, respectively why would you use them?
(Six-point scale in the range from "does not apply at all" to "fully applies")

4.1 General preparation for the OSCE
4.2 Uncertainty regarding certain practical skills
4.3 Refreshing what I have learned
4.4 General interest without specific reference to the OSCE
4.5 Preparation for clinical traineeships
4.6 Because practical skills are insufficiently taught in the curriculum

5. Other information

5.1 Did you complete vocational training in adjacent healthcare professions before starting your studies?
(yes/no)

5.2 In which adjacent healthcare profession have you completed vocational training? (Multiple responses possible)
(nursing, medical technician, paramedic, another profession)
Question only activated when the answer to 5.1 was (yes)

5.3 Have you worked in your training occupation?
(yes/no)
Question only activated when the answer to 5.1 was (yes)

5.4 Did you start or complete another degree before enrolling in your studies at MHH?
(yes/no)

5.5 Are there medical role models in your family?
(yes/no)

5.6 Are there medical role models in your circle of friends or acquaintances?
(yes/no)

Which of the following areas would you like to work in later?
(Six-point scale in the range from "very much" to "very reluctantly")

5.7 In an university hospital
5.8 In a non-university hospital
5.9 In a specials medical practice
5.10 In a general medical practice
5.11 As a researcher

5.12 In the pharmaceutical industry
5.13 In medical technology
5.14 In another field (Text box)

6. Declaration of consent

6.1 I agree that my information from this questionnaire can be linked to my examination results from the OSCE.
(yes/no)

Thank you for your participation!
(if answer to 6.1 was "yes")

Thank you for your participation. Your answers will not be linked to your OSCE results.
(if answer to 6.1 was "no")
