# Supplementary material for: Does voluntary practice improve the outcome of an OSCE in undergraduate medical studies? A Propensity Score Matching approach
Source: PLoS One. 2024 Oct 24;19(10):e0312387. doi: 10.1371/journal.pone.0312387 (PMC11500970; doi:10.1371/journal.pone.0312387)
Supplement: Attachment — Submitted filename: Responses to the Reviewers.docx [file pone.0312387.s004.docx]

**Responses to the Reviewers**

We would like to thank Dr. Saira Akhlaq and Prof. Fozia Fatima for reviewing our manuscript entitled “Does voluntary practice improve the outcome of an OSCE in undergraduate medical studies?” Below, we list our detailed responses to all comments in red script and describe how we integrated the reviewers’ suggestions into our manuscript. All changes that resulted from your suggestions are also indicated in the revised manuscript with tracked changes.

**Reviewer #1**

Reviewer #1: "The informed consent and study regulations governing the use of personal data for evaluation/research and quality assurance purposes (in particular sect. 14, para. 1-5 ‘MHH Immatrikulationsordnung’ and sect. 17, para. 3 NHG (Higher Education Act in Lower Saxony, Germany)) rendered a separate approval by an ethics committee unnecessary. The research presented here is in accordance with the Helsinki Declaration"- I do not understand this statement. Even if its exempt, you need to apply to institutional review board committee for approving the exempt status of research globally. If this is not the case in Germany-this is beyond my understanding.

As described in detail in the manuscript, the use of administrative data such as student statistics and exams is governed by the mentioned laws and university regulations, provided that the use is for the purposes of quality assurance in teaching, evaluation, or research in these areas. We ensured that the data was anonymized and that individuals in the dataset, which we submitted with the revision, are not identifiable.

In our study, the administrative data was supplemented by the voluntary participation in a short survey. We thoroughly informed the students about how and for what purpose their information and exam data would be linked and analyzed as well as about data privacy precautions. Participants had to agree explicitly to the use of their data.

The mentioned regulations were co-determined and adopted by all university committees, so additional involvement of the ethics committee is not indicated, as we did not conduct research on humans (e.g. in a clinical sense) but rather pursued research objectives and methods situated in the social sciences. In this sense, the principles formulated by the APA (“Ethical Principles of Psychologists and Code of Conduct”, American Psychologist, 2002, 57, 1060-1073) are more relevant guidelines for us. Researchers are of course free to obtain an ethics vote anyway if they wish to, but it is not required and would be redundant work. This regulation is a welcome reduction of bureaucracy, which is otherwise unfortunately very pronounced in all areas in Germany.

Incidentally, we in Germany are not alone with such regulations. In the Netherlands, studies in the field of medical education are also exempt from ethical approval (Urlings-Strop et al. (2011). Selected medical students achieve better than lottery-admitted students during clerkships. Medical Education 45: 1032-1040).

Secondly, even though ANOVA is a test of Association that can be applied when the categorical variable is non-binary. In this manuscript, to my understanding, categorical variables are binary and not non-binary.

We respectfully disagree with this comment. In ANOVA, it is perfectly acceptable to include binary variables, both as predictors (as we did in the manuscript) and as dependent variables. It is common to include binary variables, think e.g. of gender (male, female). These binary factors are just special cases of categorical variables with two levels. Including binary factors does not violate any assumptions of the ANOVA method.

Thirdly, introduction can be shortened. Even though the description about the entire OSCE is beautiful, it becomes monotonous to read about OSCE. A greater focus on the overall study variables would shorten the descriptive part of the manuscript.
Fourthly, discussion may be tightened up a little bit.

We have shortened the “Materials and Methods” section by removing parts that we did not consider central to our research question, methodological approach, and presentation of our findings. In the revised version, this section is now more focused on the essential information regarding our study variables (OSCE exam results and Skills Lab usage).

We also agree that our “Discussion” could be more tightly focused on the conceptual background of our study and the research question. Similar to the “Materials and Methods” section, we have trimmed text that we deemed non-essential for the interpretation of our results. Additionally, we have strengthened the references to the conceptual background (“assessment drives learning”) of our study in the revised manuscript and provided an outlook on a future research question.

To address Reviewer #2's request for a stronger emphasis on the conceptual background of our empirical study, we have expanded the “Introduction” with a few sentences and additional literature references. However, the overall length of our manuscript has not increased.

**Reviewer #2**

Reviewer #2: Investigation is a good topic. However, researchers must update the following.

1. The study's justification must be strengthened, and the topic of inquiry must be strengthened by a review of the literature. This study lacks a theoretical or conceptual framework that would enable readers to comprehend the genesis and processing of this notion within the context of the research issue.

We agree with that embedding any research questions and empirical analysis within the existing literature and appropriate conceptual frameworks is crucial. In our case, this is the concept of “assessment for learning” or “assessment drives learning”. In the introduction, we mentioned and describe the pillars of this framework. However, we agree that this was done very briefly. In the revised manuscript, we have expanded the explanations (in the “Introduction” and the “Discussion”) and added additional literature. To compromise with Reviewer #1’s suggestion of a shorter presentation of the introduction, methodology, and discussion, we have opted against providing an extensive literature review. Instead, we have focused on presenting the positioning of our study within the “assessment drives learning” concept and the newly added information as concisely as possible.

We would like to emphasize that our motivation for this study was not to make a conceptual contribution or to extent the “assessment for learning” framework at a theoretical level. Rather, our aim was to test the existing concept and the hypothesis (derived from it) that the anticipation of a complex practical-oral exam motivates learning efforts and targeted preparation, which should lead to better performance and competence gains. We chose the OSCE exam format because it, in conjunction with preparation in the Skills Lab, fits perfectly with the assumptions of the “assessment drives learning” concept.

Our second significant contribution is at the empirical-methodological level with the use of Propensity Score Matching (PSM). We highlighted this with the subtitle of our manuscript and through the detailed description of the method. The significant advantage of PSM is that it allows for causal inferences with observational data. To our knowledge, our study is the first to enable causal interpretations regarding the connection between preparation and performance in an OSCE outside of Randomized Controlled Trials (RCTs).

2. When it comes to methodology, the researcher needs to explain and provide support for the population, sample, sampling strategy, and sample size computation. Verify the instrument's validation as well, please.

In the “Materials and Methods” chapter, specifically in the “Data” subsection, we provide a detailed overview of the sample composition, the generation of our sample, and who our study subjects are. Regarding sample size, one unfortunately cannot control how many students participate in voluntary surveys and provide their consent. In the “Descriptive Statistics” subheading of the Results chapter, we delve into the sample details again and present figures and sample tests. Many details that we could not include in the manuscript due to space constraints are available in “Supplement II”. Additionally, we have tested and verified that our sample is representative of the entire cohorts studied; see also “Supplement I”.

The method of Propensity Score Matching (PSM), which enables the estimation of treatment effects with observational data and causal interpretations, is a focal point of our paper and represents an innovative approach to deal with observational data. Therefore, we dedicated substantial space to describing the method in the context of our research question. Furthermore, we allocated an entire subsection and a comprehensive appendix (“Supplement III”) to using state-of-the-art methods to check the robustness and validation of our results. As documented in the paper, we successfully verified the robustness of our findings.
